# Performance of loop-mediated isothermal amplification (LAMP) for the diagnosis of malaria among malaria suspected pregnant women in Northwest Ethiopia

**DOI:** 10.1186/s12936-017-1692-4

**Published:** 2017-01-19

**Authors:** Banchamlak Tegegne, Sisay Getie, Wossenseged Lemma, Abu Naser Mohon, Dylan R. Pillai

**Affiliations:** 1 Department of Medical Parasitology, School of Biomedical and Laboratory Sciences, College of Medicine and Health Sciences, University of Gondar, Gondar, Ethiopia; 2 Department of Pathology and Laboratory Medicine, Medicine, and Microbiology, University of Calgary, Calgary, AB Canada

**Keywords:** Malaria, Diagnostics, Pregnancy, Molecular, RDT

## Abstract

**Background:**

Malaria is a major public health problem and an important cause of maternal and infant morbidity in sub-Saharan Africa, including Ethiopia. Early and accurate diagnosis of malaria with effective treatment is the best strategy for prevention and control of complications during pregnancy and infant morbidity and mortality. However, laboratory diagnosis has relied on the identification of malaria parasites and parasite antigens in peripheral blood using Giemsa-stained microscopy or rapid diagnostic tests (RDTs) which lack analytical and clinical sensitivity. The aim of this study was to evaluate the performance of loop-mediated isothermal amplification (LAMP) for the diagnosis of malaria among malaria suspected pregnant women in Northwest Ethiopia.

**Methods:**

A cross sectional study was conducted from January to April 2016. Pregnant women (n = 87) suspected of having malaria at six health centres were enrolled. A venous blood sample was collected from each study subject, and analysed for *Plasmodium* parasites by microscopy, RDT, and LAMP. Diagnostic accuracy outcome measures (sensitivity, specificity, predictive values, and Kappa scores) of microscopy, RDT and LAMP were compared to nested polymerase chain reaction (nPCR) as the gold standard. Specimen processing and reporting times were documented.

**Results:**

Using nPCR as the gold standard technique, the sensitivity of microscopy and RDT was 90 and 70%, and the specificity was 98.7 and 97.4%, respectively. LAMP assay was 100% sensitive and 93.5% specific compared to nPCR.

**Conclusions:**

This study showed higher sensitivity of LAMP compared to microscopy and RDT for the detection of malaria in pregnancy. Increased sensitivity and ease of use with LAMP in point-of-care testing for malaria in pregnancy was noted. LAMP warrants further evaluation in intermittent screening and treatment programmes in pregnancy.

## Background

*Plasmodium falciparum* is the principal cause of severe malaria while *Plasmodium vivax* is increasingly recognized as capable of causing severe disease [1–4]. According to the latest World Health Organization report of 2015, there were an estimated 214 million cases of malaria worldwide with 438,000 deaths. Ninety per cent of the deaths occurred in sub-Saharan Africa where pregnant women and children are significantly affected [1].

Every year about 30 million African women in malaria-endemic areas become pregnant and are at risk of infection with malaria, and an estimated 75,000–200,000 infant deaths are reported due to malaria infection in pregnancy [5, 6]. Malaria, particularly due to *P. falciparum*, in pregnant women increases the risk of maternal death, miscarriage, stillbirth and neonatal death [7, 8]. The impact of malaria during pregnancy may vary within a country depending on the intensity of malaria transmission. In areas of seasonal malaria transmission, pregnant women are three times more likely to suffer from severe malaria as compared to non-pregnant counterparts. In areas of stable malaria, adult pregnant women would have considerable acquired immunity and infection during pregnancy typically does not cause symptomatic malaria. The effect of malaria in pregnancy is primarily low birth weight and maternal anaemia [9, 10].

The World Health Organization (WHO) recommends a three-pronged approach to reduce the burden of malaria in pregnancy: (1) provision and promotion of insecticide treated bet nets (ITN) or long-lasting insecticide- treated bed nets (LLINS); (2) administration of intermittent preventive treatment with sulfadoxine–pyrimethamine (IPTp-SP) after the first trimester of pregnancy in areas with stable malaria transmission; and (3) prompt diagnosis and appropriate treatment of malaria [5, 11]. However, because of rising *P. falciparum* resistance to SP in sub-Saharan regions, the use of rapid diagnostic tests to screen women for malaria at the first or each antenatal visit and then treat is likely more sustainable than IPTp without diagnosis [12].

Early and accurate diagnosis with effective treatment is the best strategy to decrease malaria-related pregnancy complications and infant mortality. The current malaria diagnostic methods include Giemsa-based microscopy, rapid diagnostic tests (RDTs), polymerase chain reaction (PCR) and placental histology depending on the setting [13]. The poor performance of routine malaria diagnostic techniques including RDTs and microscopy contribute to the burden of malaria in pregnant women [14, 15]. This is in large part due to the sequestration of the parasite in the placenta making the parasitaemia lower than usual in the peripheral blood. Therefore, nPCR which targets the small subunit ribosomal RNA (ssrRNA) is the better alternative diagnostic technique due to its high sensitivity (as low as 0.1 parasite/µl of whole blood). However, nPCR it is not widely used for the diagnosis of malaria in resource-limited settings as it requires a very well-equipped laboratory, and the cost of diagnosis is more expensive [16, 17].

Loop-mediated isothermal amplification (LAMP) is a simpler molecular procedure and better alternative for the field than nPCR [18, 19]. LAMP has many operational advantages over PCR including: (1) the acceptability of a crude sample preparation; (2) minimal capital equipment requirements; (3) rapid time to obtain a result; (4) lower cost; and (5) technically less complex than PCR [20].

## Methods

### Study area

The study was conducted at Kola Diba, Girargie, Aymba, Azezo, Tseda, and Sanja Health Centres, which are located in North Gondar, Amhara Regional State, Ethiopia. The district covers an area of 1270 km with a total population of about 263,000 people approximately 700 km north of Addis Ababa close to Lake Tana. The ethnic group which predominates in this region are the Amhara people. The district sits at an altitude between 1750 and 2100 m above sea level. Malaria is the most prevalent seasonal disease in the area, and is the second most common reportable disease at the health centre. Both seasonal *Plasmodium vivax* and year-long *P. falciparum* exist in the area [21, 22]. Peak malaria transmission occurs from October to December.

### Study design

A cross-sectional study was conducted among malaria suspected pregnant women at six health centres in Northwest Ethiopia from January to April (local dry season), 2016. Study participants were recruited consecutively (convenience sampling) and a total of 87 pregnant women with signs and symptoms consistent with malaria were enrolled. Pregnant women who provided the required laboratory sample for the study were included in the study. Those subjects who were severely ill according to WHO criteria and/or had received anti-malarial drugs during the past 4 weeks prior to study commencement were excluded.

### Data collection

Demographic profiles and clinical data were collected at the health centre antenatal clinic using an interview-based questionnaire translated into the vernacular language that the participant could understand. In addition, two milliliters of venous blood was collected from each study participant using an EDTA anti-coagulated test tube. Soon after venous blood collection, a drop of blood was taken for the RDT, then two separate drops of blood were placed on a frosted microscopic glass slide to prepare both thin and thick blood films, and another two separate drops of blood was placed on Whatman filter for nPCR. The rest of the blood was kept at 2–8 °C at the health centre then transported to the Medical Parasitology Laboratory, University of Gondar on weekly basis to be kept frozen until analysed with LAMP. Blood films were processed, and parasite detection and parasitaemia level was determined according to WHO standards [23]. CareStart™ HRP2/PLDH COMBO (Pf/Pan) detection kits were used for RDT testing. CareStart™ Pf/Pan targets histidine rich protein 2 for *P. falciparum* and lactate dehydrogenase for the diagnosis of non-falciparum species.

### LAMP process and detection of malaria parasites

Loopamp™ malaria Pan/Pf detection kits (Eiken Chemicals, Tokyo, Japan) which detect mitochondrial DNA were used to amplify *Plasmodium/P. falciparum* DNA. The preparation of the LAMP mixture, sample running and detection was performed as described previously [24]. Briefly, the parasite DNA was extracted by a “boil and spin” method where 60 μl of EDTA blood was added to 60 μl of extraction solution (400 mM NaCl, 40 mM Tris pH 6.5, and 0.4% sodium dodecyl sulfate) in an Eppendorf tube, heated for 5 min at 95 °C with a water bath, then centrifuged at 10,000× *g* for 3 min. The supernatant (30 ml) was pipetted into a dilution tube containing 345 μl of sterile water, and finally 30 μl of diluted DNA sample was used in the LAMP assay. Reaction tubes containing extracted DNA sample were incubated at 65 °C in a water bath for 40 min. Amplification was detected by naked eye based on turbidity. Two additional blinded laboratory technicians read the turbidity observed at the end of the LAMP reaction for the final consensus result.

### Nested PCR process and detection of malaria parasites

Genomic DNA was extracted from dried blood on Whatman filter paper 903 (GE Healthcare) and nPCR analysis was performed as described previously [25].

### Data analysis

Data were entered into Microsoft Excel program and then analysed with SPSS version 20. Sensitivity, specificity, predictive values and Cohen’s kappa coefficient were determined using SISA online statistical software [26]. All discordant specimens were repeated by LAMP and/or nPCR.

## Results

A total of 87 malaria suspected pregnant women with the mean (SD) age of 27.43 (+5.2) years were enrolled into the study, of which 50.6% (45/87) had a previous malaria history (Table 1). The majority (52.9%) of patients were in the 25–30 age group. Second (41.4%) and third trimester (41.4%) pregnancies were more common than first trimester (17.2%). Close to three quarters (74.7%) of patients were multigravida. The overall malaria positivity rate among the study participants by nPCR was 11.5% (10/87). Ten malaria positive patients (11.5%) were detected by Giemsa microcopy with an overall median parasitaemia density of 3380/μl (parasitaemia range 400–24,760/μl). Five of the Giemsa microscopy positives were *P. falciparum* (parasitaemia density minimum/maximum: 400/7840 per μl); three were *P. vivax* (parasitaemia density minimum/maximum: 520/24,760 per μl), and two were mixed infections of *P. falciparum* and *P. vivax* (parasitaemia density minimum/maximum: 520/5360 per μl). LAMP identified 15 positive (17.2%) specimens and RDT 9 (10.3%) positives. A higher positivity rate of both single and mixed *Plasmodium* species infection were reported by LAMP than other diagnostic techniques used in this study (Table 2).

**Table 1:** Demographic and clinical data of the study particpants

Variable	Frequency^a^		Pearson Chi Square (*p* value)
	Positive: N (%)	Negative: N (%)	
Age			
18–24	3 (13.0)	20 (87.0)	1.05 (0.789)
25–30	5 (10.9)	41 (89.1)	
31–35	1 (7.1)	13 (92.9)	
36+	1 (25.0)	3 (75.0)	
Previous malaria history			
Yes	5 (11.4)	39 (88.6)	0.001 (0.969)
No	5 (11.6)	38 (88.4)	
Trimester			
First	2 (13.3)	13 (86.7)	0.61 (0.74)
Second	5 (13.9)	31 (86.1)	
Third	3 (8.3)	33 (91.7)	
Gravid			
Primigravidae	2 (9.1)	20 (90.9)	0.167 (0.683)
Multigravidae	8 (12.3)	57 (87.7)	

^a^Parasite positivity is determined by nPCR

**Table 2:** Malaria positivity rate by diagnostic techniques among the study participants

Results	Diagnostic tool			
	Microscopy	RDTs	LAMP	nPCR
Pf	5	3	5	7
Pv	3	3	5	2
Pf + Pv	2	3	5	1
Total positives	10	9	15	10
Total negatives	77	78	72	77

Using nPCR as the gold standard, LAMP had the highest sensitivity (100%; 95% CI 100) compared to microscopy (90%; 95% CI 66.3–113.7) and RDT (70%; 95% CI 33.8–106.2). Microscopy had the greatest specificity (98.7%; 95% CI 96.5–101.9) compared to RDT (97.4%; 95% CI 92.9–101.9) and LAMP (93.5%; 95% CI 86.5–100.5). There were five discordant results between nPCR and LAMP where five of them were negative by nPCR but positive by LAMP. There was also one study participant with a parasitaemia load of 520/μl negative only by nPCR. In this study, LAMP showed better sensitivity but lower specificity than microscopy and RDTs using nPCR as the gold standard (Table 3). Discordant results are itemized in Table 4. RDTs had the quickest turnaround time at 23 min compared to LAMP (60 min), microscopy (60 min), and nPCR (130 min) (Table 5). Both RDT and LAMP can be performed at the point of care (community-based testing) without the need for laboratory facilities.

**Table 3:** Performance characteristics of Microscopy, RDTs and LAMP compared to nPCR for the diagnosis of malaria among study participants

Tools	Sensitivity % (95% CI)	Specificity % (95% CI)	PPV %	NPV %	Kappa value
Microscopy	90 (66.3–113.7)	98.7 (96.5–101.9)	90	98.7	0.887
RDTs	70 (33.8–106.2)	97.4 (92.9–101.9)	77.8	96.2	0.705
LAMP	100 (100)	93.5 (86.5–100.5)	66.7	100	0.768

**Table 4:** Discordant analysis for microscopy, RDT, LAMP and nPCR for malaria diagnosis in malaria suspected pregnant women

Results	Diagnostic tools					
	RDT, Microscopy and nPCR (−), LAMP (+)	RDT, Microscopy and LAMP (+), nPCR (−)	RDT and Microscopy (−), LAMP and nPCR (+)	nPCR, LAMP and Microscopy (+), RDT (−)	nPCR LAMP and Microscopy (−), RDT (+)	Negative by four methods
*Pf*	2	1	1	1	1	
*Pv* (pan)	3					
*Pf, Pv* (pan)						
Negatives						71

*Pan* all species of malaria except *P. falciparum* (LAMP and RDT readouts do not discriminate between non-*falciparum* species)

*+* Indicates positive results,*−* indicates negative results, *Pf P. falciparum*, *Pv P. vivax*

**Table 5:** Total turnaround time required for the diagnosis of malaria with diagnostic tools among study participants at Kola Diba health center

Diagnostic tools	Time in minute from sample preparation to reporting		
	Sample preparation (min)	Testing and reporting (min)	Total time (min)
RDTs	2	21	23
Microscope	40	20	60
LAMP	5	55	60
nPCR	60	250	310

## Discussion

Pregnant women have an increased susceptibility to infection by *Plasmodia* spp. Parasites sequestered in the placenta are sometimes not detectable in peripheral blood smears by Giemsa microscopy [14]. Infection can result in maternal anaemia, prematurity and intrauterine growth retardation (IUGR) and infant low birth weight (LBW) [6]. A study from Cameroon revealed that 20.9% of pregnant women who had placental malaria were negative by peripheral blood smear [27].

Malaria remains a leading cause of morbidity and mortality especially among pregnant women and children in the developing world [1]. In the current study, the rate of malaria positivity was lower than a similar study conducted in Cameroon which had 21.9% positivity by microscopy [27]; Ghana, 19, 34, and 53% positivity by microscopy, RDTs and PCR, respectively [28]; Nigeria, 27 and 30% positivity by RDTs and microscopy, respectively [29]; Mozambique, 18.7, 15.4 and 44.8% positivity by RDTs, microscopy and quantitative PCR, respectively [14]. The discrepancy in positivity may be due to the seasonality of transmission levels in Ethiopia. The rate of malaria may vary depending on the district, intensity of malaria transmission, season, density of parasitaemia, immunity level acquired, administration of malaria chemoprophylaxis, and diagnostic methods used [27, 30–33]. Although the current study area was reported to be endemic to malaria, its transmission was low during the dry season. Of note, malaria transmission has decreased over time in this region presumably due to the introduction of WHO-endorsed control strategies except the deployment of intermittent preventive therapy [21].

In the current study, age group, trimester and parity were not statistically associated with malaria infection. Based on nPCR results, seventy percent (7/10) of malaria infected pregnant women were multigravida. In contrast, a study from Gabon revealed that primigravida and young pregnant women were associated with increased malaria susceptibility. Although not statistically significant, based on microscopy results, primigravida women demonstrated a higher median parasite density (5200 parasites/μl) than multigravida (1560 parasites/μl) [34]. This is similar to a report from Nigeria, where primigravida women demonstrated higher parasitaemia than multigravida women [35].

Polymerase chain reaction (PCR) in its various formats has emerged as the most sensitive method able to detect low levels of parasites in the blood especially in this setting [36, 37]. However, PCR requires high capital investment costs, service agreements, reagent supplies, and trained staff in molecular technologies with robust quality assurance programmes. LAMP while being molecular in nature permits crude, easy-to-perform DNA extraction and visual detection even possible in the field. Previous studies from Ethiopia and Thailand revealed that a rapid and user friendly LAMP had comparable performance to nPCR for the diagnosis of malaria in the general population [19, 24]. The current study also indicated that LAMP had better sensitivity (100%) than RDTs (70%) and Giemsa microscopy (90%) for the diagnosis of malaria in pregnant women. Increased sensitivity is essential to the malaria eradication campaign especially in populations such as pregnant women where low levels of infection go undetected.

The current study showed lower specificity of LAMP (93.5%) than RDTs and Giemsa microscopy. This result was in line with a study conducted in Bangladesh where LAMP revealed lower specificity (58.3%) than microscopy and RDT (both 100%) when compared with nPCR [18]. In the current study, there were five discordant results between LAMP and nPCR in which five samples positive by LAMP ended up negative by nPCR. This result was similar to a study conducted in Thailand where four LAMP positive results were negative by nPCR [38]. These authors suggested the need for a more sensitive PCR technique to accurately evaluate the performance of LAMP. Indeed, the role of nPCR as the *bona fide* gold standard could be questioned in this study as it relied on filter paper samples which may be subject to DNA degradation in transport to Canada from Ethiopia. DNA degradation on filter paper may ultimately explain the lower kappa value between nPCR and LAMP versus nPCR and microscopy. In support of this supposition, one specimen was positive by all three methods except nPCR. Thus, additional LAMP positive cases may be true positives and warrant treatment. However, others have reported that genomic DNA is stable on filter papers over time making this less likely a contributing factor to the discordance observed [39]. All discordant specimens were repeated by nPCR and/or LAMP to confirm the discrepancy. It is also possible that LAMP resulted in false positive amplification due to contamination as observed previously in this setting [24]. To limit this confusion, future studies should attempt LAMP and reference PCR on the same specimen at the field site to remove the confounder of filter paper versus fresh blood.

In the current study, two samples which were negative by RDTs ended up positive by LAMP, Giemsa microscopy and nPCR. For accurate diagnosis of malaria with RDTs, at least 100–500 parasites/μl of whole blood are required in peripheral blood. This was supported by a study from Tanzania which revealed that lower density of malaria parasitaemia is highly associated with the negative result of RDTs [32]. In the present study, malaria parasitaemia level in these two samples was 400 and 640 parasites/μl. However, the performance of RDTs could be also be affected by incorrectly reading faint positive or invalid results as negative [40]. There was one sample which was positive for *P. falciparum* only by RDTs. Histidine rich protein-2 (HRP-2) antigen could persist for a long time even after effective treatment, giving false positive RDT test results in the absence of active *P. falciparum* infection [41].

## Conclusions

Pregnant women are more vulnerable to malaria, harbour low level infections, and suffer malaria related complications themselves and to the neonate. It is important to use a highly sensitive, field-friendly detection method [41]. LAMP with its superior sensitivity to traditional lateral flow RDTs has the potential to replace RDTs and improve detection of *Plasmodium* parasites in intermittent screening programmes during pregnancy.

## Abbreviations

RDT: rapid diagnostic test
LAMP: loop mediated isothermal amplification

## References

[1] WHO. World malaria report 2015. Geneva: World Health Organization; 2015.

[2] Strydom AK, Ismail F, Frean J (2014). *Plasmodium ovale*: a case of not-so-benign tertian malaria. Malar J..

[3] Zubairi AB, Nizami S, Raza A, Mehraj V, Rasheed AF, Ghanchi NK (2013). Severe *Plasmodium vivax* malaria in Pakistan. Emerg Infect Dis.

[4] Rahimi BA, Thakkinstian A, White NJ, Sirivichayakul C, Dondorp AM, Chokejindachai W (2014). Severe vivax malaria: a systematic review and meta-analysis of clinical studies since 1900. Malar J..

[5] Marchesini P, Crawley J. Reducing the burden of malaria in pregnancy. MERA IV, supporting agency–Roll Back Malaria, World Health Organization; 2004.

[6] Steketee RW, Nahlen BL, Parise ME, Menendez C (2001). The burden of malaria in pregnancy in malaria-endemic areas. Am J Trop Med Hyg.

[7] De Beaudrap P, Turyakira E, White LJ, Nabasumba C, Tumwebaze B, Muehlenbachs A (2013). Impact of malaria during pregnancy on pregnancy outcomes in a Ugandan prospective cohort with intensive malaria screening and prompt treatment. Malar J..

[8] Luxemburger C, McGready R, Kham A, Morison L, Cho T, Chongsuphajaisiddhi T (2001). Effects of malaria during pregnancy on infant mortality in an area of low malaria transmission. Am J Epidemiol.

[9] WHO/UNICEF. The Africa malaria report 2003. Geneva: World Health Organization; 2003.

[10] Newman RD, Hailemariam A, Jimma D, Degifie A, Kebede D, Rietveld AE (2003). Burden of malaria during pregnancy in areas of stable and unstable transmission in Ethiopia during a non-epidemic year. J Infect Dis.

[11] Pell C, Meñaca A, Afrah NA, Manda-Taylor L, Chatio S, Were F (2013). Prevention and management of malaria during pregnancy: findings from a comparative qualitative study in Ghana, Kenya and Malawi. Malar J..

[12] Van Eijk AM, Hill J, Noor AM, Snow RW, Ter Kuile FO (2015). Prevalence of malaria infection in pregnant women compared with children for tracking malaria transmission in sub-Saharan Africa: a systematic review and meta-analysis. Lancet Glob Health..

[13] Fried M, Muehlenbachs A, Duffy PE (2012). Diagnosing malaria in pregnancy: an update. Expert Rev Anti Infect Ther..

[14] Mayor A, Moro L, Aguilar R, Bardaj A, Cistero P, Serra-Casas E (2012). How hidden can malaria be in pregnant women? Diagnosis by microscopy, placental histology, polymerase chain reaction and detection of histidine-rich protein 2 in plasma. Clin Infect Dis.

[15] Tangpukdee N, Duangdee C, Wilairatana P, Krudsood S (2009). Malaria diagnosis: a brief review. Korean J Parasitol.

[16] WHO. Malaria diagnostics technology and market landscape. 2nd ed. Geneva: World Health Organization; 2014.

[17] Mens PF, van Amerongen A, Sawa P, Kager PA (2008). Schallig DFH. Molecular diagnosis of malaria in the field: development of a novel 1-step nucleic acid lateral flow immunoassay for the detection of all 4 human Plasmodium spp. and its evaluation in Mbita, Kenya. Diagn Microbiol Infect Dis.

[18] Paris DH, Imwong M, Faiz AM, Hasan M, Yunus EB, Silamut K (2007). Loop-mediated isothermal PCR (LAMP) for the diagnosis of falciparum malaria. Am J Trop Med Hyg.

[19] Pöschl B, Waneesorn J, Thekisoe O, Chutipongvivate S, Panagiotis K (2010). Comparative diagnosis of malaria infections by microscopy, nested PCR, and LAMP in northern Thailand. Am J Trop Med Hyg.

[20] Dhama K, Karthik K, Chakraborty S, Tiwari R, Kapoor S (2014). Loop-mediated isothermal amplification of DNA (LAMP): a new diagnostic tool lights the world of diagnosis of animal and human pathogens: a review. Pak J Biol Sci.

[21] Alemu A, Muluye D, Mihret M, Adugna M, Gebeyaw M (2012). Ten year trend analysis of malaria prevalence in Kola Diba, North Gondar, Northwest Ethiopia. Parasit Vectors..

[22] Alemu A, Fuehrer HP, Getnet G, Tessema B, Noedl H (2013). *Plasmodium ovale curtisi* and *Plasmodium ovale wallikeri* in North–West Ethiopia. Malar J..

[23] WHO. Methods for surveillance of antimalarial drug efficacy. Geneva: World Health Organization; 2009.

[24] Sema M, Alemu A, Bayih AG, Getie S, Getnet G, Guelig D (2015). Evaluation of non-instrumented nucleic acid amplification by loop-mediated isothermal amplification (NINA-LAMP) for the diagnosis of malaria in Northwest Ethiopia. Malar J..

[25] Snounou G, Viriyakosol S, Zhu XP, Jarra W, Pinheiro L, do Rosario VE (1993). High sensitivity of detection of human malaria parasites by the use of nested polymerase chain reaction. Mol Biochem Parasitol.

[26] SISA online statistical software www.quantitativeskills.com/sisa/.

[27] Leke RF, Djokam RR, Mbu R, Leke RJ, Fogako J, Megnekou R (1999). Detection of the *Plasmodium falciparu*m antigen histidine-rich protein 2 in blood of pregnant women: implications for diagnosing placental malaria. J Clin Microbiol.

[28] Mockenhaupt FP, Bedu-Addo G, von Gaertner C, Boyé R, Fricke K, Hannibal I (2006). Detection and clinical manifestation of placental malaria in southern Ghana. Malar J..

[29] Umeh SI, Enwuru CP, Egbuobi RC (2013). Diagnosis of malaria in pregnancy: a comparison of microscopy with rapid diagnostic tests. Microbiol Res Int.

[30] Briand V, Cottrell G, Massougbodji A, Cot M (2007). Intermittent preventive treatment for the prevention of malaria during pregnancy in high transmission areas. Malar J..

[31] Schlagenhauf P, Petersen E (2008). Malaria chemoprophylaxis: strategies for risk groups. Clin Microbiol Rev.

[32] Eckhoff PA (2012). Malaria parasite diversity and transmission intensity affect development of parasitological immunity in a mathematical model. Malar J..

[33] Ishengoma DS, Francis F, Mmbando BP, Lusingu JPA, Magistrado P (2011). Accuracy of malaria rapid diagnostic tests in community studies and their impact on treatment of malaria in an area with declining malaria burden in north-eastern Tanzania. Malar J..

[34] Bouyou-Akotet MK, Ionete-Collard DE, Mabika-Manfoumbi M, Kendjo E, Matsiegui PB, Mavoungou E (2003). Prevalence of *Plasmodium falciparum* infection in pregnant women in Gabon. Malar J..

[35] Raimi O, Kanu C (2010). Prevalence of malaria infection in pregnant women living in a suburb of Lagos. Afr J Biochem Res..

[36] Alemu A, Fuehrer HP, Getnet G, Kassu A, Getie S, Noedl H (2014). Comparison of Giemsa microscopy with nested PCR for the diagnosis of malaria in North Gondar, north-west Ethiopia. Malar J..

[37] Getnet G, Getie S, Srivastava M, Birhan W, Fola AA, Noedl H (2015). Diagnostic performance of rapid diagnostic tests for the diagnosis of malaria at public health facilities in north-west Ethiopia. Trop Med Int Health..

[38] Ocker R, Prompunjai Y, Chutipongvivate S, Karanis P (2016). Malaria diagnosis by loop-mediated isothermal amplification (LAMP) in Thailand. Rev Inst Med Trop São Paulo..

[39] Chaisomchit S, Wichajarn R, Janejai N, Chareonsiriwatana W (2005). Stability of genomic DNA in dried blood spots stored on filter paper. Southeast Asian J Trop Med Public Health.

[40] Counihan H, Harvey SA, Sekeseke-Chinyama M, Hamainza B, Banda R, Malambo T (2012). Community health workers use malaria rapid diagnostic tests (RDTs) safely and accurately: results of a longitudinal study in Zambia. Am J Trop Med Hyg.

[41] Kyabayinze DJ, Tibenderana JK, Odong GW, Rwakimari JB, Counihan H (2008). Operational accuracy and comparative persistent antigenicity of HRP2 rapid diagnostic tests for *Plasmodium falciparum* malaria in a hyperendemic region of Uganda. Malar J..

